# Clopidogrel-Associated Interstitial Lung Disease: A Case Report and Literature Review

**DOI:** 10.7759/cureus.28394

**Published:** 2022-08-25

**Authors:** Sheeraz Abro, Viktoriya Bikeyeva, Warda A Naqvi, Chinyere L Anigbo, Farhan Tariq, Ramish Hussain Rafay, Muhammad Faiq Umar, Romil Singh

**Affiliations:** 1 Internal Medicine, Chandka Medical College, Larkana, PAK; 2 Internal Medicine, California Institute of Behavioral Neurosciences & Psychology, Fairfield, USA; 3 Infectious Diseases, Shifa International Hospital Islamabad, Islamabad, PAK; 4 Internal Medicine, University of Nigeria, Enugu, NGA; 5 Medicine, Dow University of Health Sciences, Dow International Medical College, Karachi, PAK; 6 Internal Medicine, Mayo Hospital, Lahore, PAK; 7 Medicine, Mayo Hospital, Lahore, PAK; 8 Critical Care, Allegheny Health Network, Pittsburgh, USA

**Keywords:** drug-induced interstitial lung diseases, clopidogrel hypersensitivity, cardiovascular drug, interstitial lung disease, clopidogrel

## Abstract

Clopidogrel is an antithrombotic agent widely used for the secondary prevention of cerebrovascular and cardiovascular complications. Clopidogrel can cause serious adverse events, including gastrointestinal bleeding. Pulmonary complications caused by clopidogrel are not widely described, and clopidogrel-induced interstitial lung disease (ILD) is rare. Here, we report a case of drug-induced ILD in a patient who presented with dyspnea, chest pain, and mild fever. The patient underwent percutaneous coronary intervention two months ago and was commenced on clopidogrel. He was diagnosed with clopidogrel-induced ILD based on clinical and imaging findings, history of drug exposure without any change, exclusion of other respiratory disorders, and clinical improvement after discontinuation of clopidogrel and steroid use.

## Introduction

Clopidogrel belongs to a class of thienopyridine drugs that inhibit the platelet aggregation and action of adenosine phosphate resulting in increased bleeding time and reduced blood viscosity [1]. Clopidogrel is widely used for reducing the risk of fatal thrombosis and the secondary prevention of complications in ischemic stroke and myocardial infarction [2]. Similar to other cardiovascular drugs such as aspirin and ticlopidine, clopidogrel has several complications, and the most common adverse event is gastrointestinal bleeding [3]. Pulmonary complications after clopidogrel use have not been widely reported. A few cases have been discussed in the literature [4,5]. Here, we report a case of interstitial lung disease (ILD) induced by clopidogrel after two months of the administration.

## Case presentation

A 59-year-old male was brought to the emergency department with worsening shortness of breath for the last five days. He also complained of mild fever, bilateral chest pain, anorexia, and nausea. He reported no rhinorrhea, cough, sputum, and rigors or chills. He was diagnosed with non-ST-elevation myocardial infarction (NSTEMI) two months ago and underwent percutaneous coronary intervention. Subsequently, he was commenced on 75 mg clopidogrel daily, 100 mg aspirin daily, and 40 mg atorvastatin daily. He complied with his medications and had no history of smoking, alcohol abuse, and illicit drug use. He also reported no history of orthopnea, leg swelling, allergic reactions, autoimmune diseases, or exposure to pets or humidifiers.

**Figure 1:**
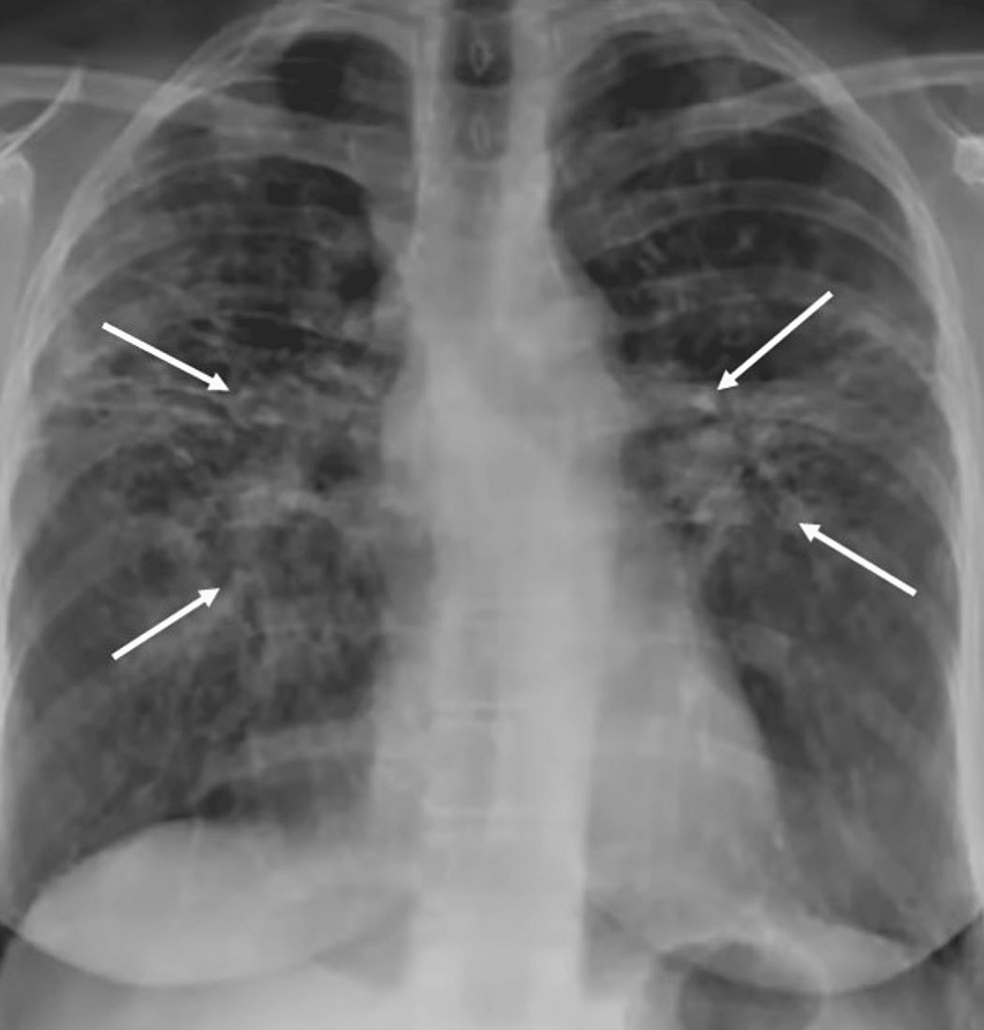
Chest X-ray demonstrating scattered ground-glass opacities and multifocal consolidation.

On initial evaluation, he was febrile (100.5°F), with a heart rate of 110 beats/minute, respiratory rate of 32 breaths/minute, blood pressure of 130/80 mmHg, and oxygen saturation of 86% on room air. Physical examination did not reveal any lymphadenopathy or skin rash. There were coarse crackles in both lungs on auscultation. His repeat coronavirus disease 2019 (COVID-19) polymerase chain reaction (PCR) test was negative on multiple occasions. A chest X-ray showed scattered ground-glass opacities (GGOs) and multifocal consolidation (Figure 1). The results of his initial laboratory test are shown in Table 1. Initial arterial blood gas analysis demonstrated normal acid-base balance and normal anion gap.

**Table 1:** Results of initial laboratory tests. LDH: lactate dehydrogenase; WBC: white blood cell; ALT: alanine aminotransferase; CRP: C-reactive protein; RBC: red blood cell; BUN:  blood urea nitrogen; AST: aspartate aminotransferase; ESR: erythrocyte sedimentation rate; ALP: alkaline phosphatase

Parameter	Lab value (reference range)
Hemoglobin	11.9 (12–16.5) g/dL
RBC count	3.9 (4.2–5.4) million cells/µL
WBC count	8100 (4,000–11,000)/µL
Platelet count	275,000 (150,000–450,000)/µL
LDH	202 (105–333) IU/L
ESR	18 (1–13) mm/hour
ALT	45 (<40) IU/L
AST	51 (<35) IU/L
ALP	202 (<240) IU/L
D-dimer	0.21 (<0.50) mg/L
CRP	03 (<10) mg/L
BUN	21 (6–20) mg/dL
Serum creatinine	1.1 (0.7–1.3) mg/dL

**Figure 2:**
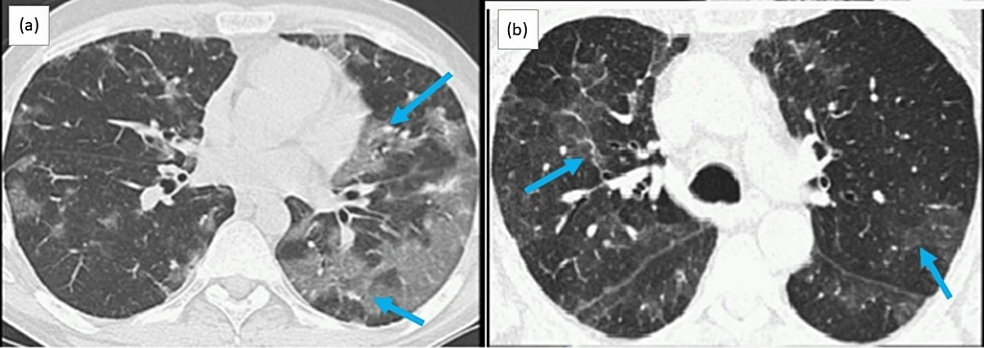
High-resolution computed tomography of the chest demonstrating diffuse crazy-paving and consolidation bilaterally (a) and focal parenchymal band in both lungs (b).

He was commenced on broad-spectrum antibiotics with a provisional diagnosis of pneumonia. He underwent high-resolution computed tomography (HRCT) of the chest, which revealed diffuse crazy-paving, diffuse dense consolidation bilaterally, and a focal parenchymal band in both lungs (Figure 2).

The patient remained febrile the following day, and his supplemental oxygen demand increased. He underwent diagnostic bronchoscopy, and bronchoalveolar lavage fluid (BALF) samples demonstrated clear-colored fluid. Bacterial blood and BALF culture, acid-fast bacilli smear, and viral PCR for cytomegalovirus were negative from BALF samples, and no organism was detected. Clopidogrel-induced ILD was suspected because no other additional factors were identified. His dual antiplatelet regimen was changed to aspirin only, and he was commenced on intravenous 1 mg/kg/day methylprednisolone. He was discharged with prednisolone 25 mg/day 12 days later when improvements in his clinical manifestations and chest X-ray were observed. The prednisolone was gradually tapered over two months.

## Discussion

ILD is an umbrella term used for a large group of disorders that cause lung scarring or fibrosis. Lung scarring causes stiffness in the lungs resulting in breathing difficulty. Lung injury from ILD is often irreversible and worsens over time [6]. ILD can be idiopathic, and this variant is the most common type of ILD. Other etiologies that lead to ILD include autoimmune disease, chemotherapy, radiation, drugs, and environmental and occupational exposure [6]. Prevalence of ILD is 20% higher in men (80.9 per 100,000) than in women (67.2 per 100,000), and data demonstrated that 2.5-3% of ILD is induced by drugs. Antitumor drugs are the leading cause of ILD (23-52%), followed by antirheumatic drugs, antibiotics, and antiarrhythmic drugs [7]. Pulmonary complications of antiplatelet agents include alveolar hemorrhage and ILDs such as organizing pneumonia or eosinophilic pneumonia. The reported cases of clopidogrel-induced pulmonary complications are highlighted in Table 2 [4,5,8-11].

**Table 2:** Cases of lung injury induced by clopidogrel use. M: male; ILD: interstitial lung disease; F: female

Authors	Antiplatelet drug	Age/sex	Reason for antiplatelet use	Duration of drug use	Diagnosis	Management
An et al. [4]	Clopidogrel	79/F	Cerebral infarction	Two weeks	ILD	Discontinue clopidogrel, steroids
Tomoda et al. [5]	Clopidogrel	75/M	Acute coronary syndrome	Three months	ILD	Discontinue clopidogrel, steroids
Kim et al. [8]	Clopidogrel	62/M	Myocardial infarction	Three days	Alveolar hemorrhage	Discontinue clopidogrel
Kilaru et al. [9]	Clopidogrel	56/M	Myocardial infarction	Not reported	Alveolar hemorrhage	Discontinue clopidogrel
Erdinler et al. [10]	Clopidogrel	71/M	Carotid stenosis	Two days	ILD, pleural effusion	Antibiotics, discontinue clopidogrel
Sarrot-Reynauld et al. [11]	Clopidogrel	57/M	Myocardial infarction	Five days	ILD	Discontinue clopidogrel

The pathophysiology of drug-induced ILD is not well understood; however, it seems that allergic reactions and neutrophil activation may lead to lung scarring and fibrosis instead of direct toxicity from the drugs or chemicals [7,12]. It has been reported that allergic reactions to drugs may cause ILD because discontinuation of the offending agent and use of corticosteroids resulted in remission [8-10]. Additionally, clopidogrel may also activate neutrophils and macrophages, leading to fibrosis and scarring, proving evidence of pathogenesis of lung injury [7,13]. Because data regarding drug-related immune response is limited, more studies are warranted to understand the pathophysiology of drug-induced initiation and progression of ILD.

Diagnosis of ILD requires hematological, immunological, and radiological workup, including HRCT. Because chest X-ray is less accurate in detecting early degenerative changes, it is usually followed by HRCT of the chest, which is the investigation of choice for detecting ILD [13]. Spirometry can also be done to identify the type of respiratory abnormality (restrictive/obstructive) and assess the diffusion capacity of the lungs. However, to obtain a definitive diagnosis of pulmonary fibrosis, a microscopic examination of the lung tissue obtained from bronchoscopy, bronchoalveolar lavage, or surgical biopsy is necessary [13]. If untreated, ILD can lead to a series of life-threatening complications, including pulmonary hypertension, right-sided heart failure, respiratory failure, and secondary infections. ILD is managed by treating the underlying etiology and corticosteroids [14].

Our patient presented with dyspnea, chest pain, and mild fever. He was diagnosed with clopidogrel-induced ILD based on clinical and imaging findings, history of drug exposure without any change, exclusion of other respiratory disorders, and clinical improvement after clopidogrel discontinuation and steroid use. The only limitation of our case is that our diagnosis was not based on histopathological results.

## Conclusions

Our case highlights that patients taking clopidogrel may develop drug-induced ILD, which is a concerning finding given the widespread clopidogrel use as an antiplatelet agent. Although clopidogrel-induced ILD is uncommon, it should be included among the differential causes of drug-induced ILD. ILD identification is challenging because of non-specific clinical, radiological, and histological findings. Therefore, physicians must acknowledge the possible associations between the drugs and ILD. Further studies are also warranted to explain the unclear mechanisms of clopidogrel-induced ILD.
